# The role of miRNAs in cutaneous squamous cell carcinoma

**DOI:** 10.1111/jcmm.12649

**Published:** 2015-10-27

**Authors:** Xin Yu, Zheng Li

**Affiliations:** ^1^ Department of Orthopaedic SurgeryPeking Union Medical College HospitalChinese Academy of Medical Sciences and Peking Union Medical CollegeBeijingChina

**Keywords:** cutaneous squamous cell carcinoma, microRNAs, miR, oncogene

## Abstract

MicroRNAs (miRs) are small, noncoding RNAs that negatively regulate gene expressions at posttranscriptional level. Each miR can control hundreds of gene targets and play important roles in various biological and pathological processes such as hematopoiesis, organogenesis, cell apoptosis and proliferation. Aberrant miR expression contributes to initiation and cell progression of cancers. Accumulating studies have found that miRs play a significant role in cutaneous squamous cell carcinoma (cSCC). Deregulations of miRs may contribute to cSCC carcinogenesis is through acting as oncogenic or tumour suppressive miRs. In this study, we summarized the recent data available on cSCC‐associated miRs. In particular, we will discuss the contribution of miR to the initiation and progression of cSCCs. Although there are many obstacles to be overcome, clinical use of miRs as biomarkers for diagnosis, prediction of prognosis and target for therapies, will be a promising area in the future with more expression and functional role of miRs revealed.

## Introduction

Cutaneous squamous cell carcinoma (cSCC) is the second most common skin cancer among Caucasian population, with a constantly increasing incidence estimated to 700,000 new cases diagnosed each year in the USA alone 1, 2. Cutaneous squamous cell carcinoma is derived from epidermal keratinocyte, which is more malignant than basal cell cancers because cSCC grow and spread much faster 3, 4. Cutaneous squamous cell carcinoma commonly develops on sun‐exposed areas of the body since ultraviolet light (UV) exposure and immunosuppression are the major risk factors for the development of cSCC 5. Most cSCCs are considered to be low risk cancers and are usually cured by surgery and/or radiotherapy. However, their potential to recur and metastasize often raises therapeutic problems 6. For patients with metastatic cSCC, the long‐term prognosis is poor, with a 1‐year disease‐specific survival at 44–56% 7. Therefore, it is urgent to develop more effective targets or early detection and new therapeutic approaches.

MicroRNAs (miRs) are small, non‐coding RNAs, which can negatively regulate gene expression at the post‐transcriptional level by binding to the target genes 8, 9. MicroRNA participate in many physiological and pathologic cellular functions, regulating cell cycle, differentiation, development and metabolism 10, 11. In addition, deregulation of miR expression has been shown to play significant roles in a variety of cancers, in which miRs can act as tumour suppressors or oncogenes by regulating the multiple target genes 8, 12. Recently, miR deregulation has been proved to be involved in the development of cSCC (Table 1).

**Table 1:** Deregulated miRNAs in cSCCs and their target genes

Expression changes	MiRNAs	References	Role	Target genes
Downregulated	miR‐1	17, 22	Suppressive	Met; Twf1; Ets1; Bag4
	miR‐34a	24	Suppressive	SIRT6
	miR‐124/214	32	Suppressive	ERK2
	miR‐125b	39	Suppressive	MMP13
	miR‐155	49	Suppressive	Not mentioned
	miR‐193b/365a	54	Suppressive	KRAS; MAX
	miR‐199a	60	Suppressive	CD 44
	miR‐361‐5p	55	Suppressive	VEGFA
	miR‐483‐3p	68	Suppressive	API5; BIRC5; RAN
Up‐regulated	miR‐21	39, 75, 76, 77	Promotive	PDCD4; GRHL3; PTEN
	miR‐31	39, 82	Promotive	Not mentioned
	miR‐205	83	Promotive	SHIP2
	miR‐365	85, 86	Promotive	NFIB

NFIB: nuclear factor I/B; cSCCs: cutaneous squamous cell carcinomas; MMP13: matrixmetallopeptidase 13.

In this review, we will discuss the miRs involved in the cSCCs development. In addition, we will also discuss their potential implications in the creation of novel diagnostic tools and promising future therapies.

## Deregulated microRNAs in cSCC

### MiRs down‐regulated in cSCC

#### MiR‐1

MicroRNA‐1, a 22‐nucleotide miR, might be a tumour suppressor since it is significantly decreased in multiple types of cancers including skin, lung, liver, bladder, renal and prostate cancer 13, 14, 15, 16, 17. In addition, increased expression of miR‐1 inhibits cell migration and growth while it promotes apoptosis *in vitro*. MicroRNA‐1 participates in some classic oncogenic signalling pathways such as those controlled by Met, Cyclin D, Wnt, FOXP1 and TAGLN2 13, 16, 18, 19, 20, 21.

Fleming *et al*. found that miR‐1 expression was frequently reduced both in mouse cSCCs and murine cSCC cell lines 22. Furthermore, functional studies showed that miR‐1 inhibited proliferation and increased apoptosis in cSCC cell line. Met, Twf1 and Ets1 and Bag4 were identified as the targets of miR‐1, indicating that miR‐1 could target various genes to suppress cSCC development. MicroRNA‐1, along with miR‐133a, miR‐205 and let‐7d was downregulated in SCCs of head and neck (HNSCC). Moreover, miR‐1 significantly decreased HNSCC cell proliferation, invasion and migration, and promoted cell apoptosis and cell cycle arrest 17.

Overall, the role for miR‐1 in the regulation of proliferation, invasion, metastasis and apoptosis in cSCC suggest its potential application as therapeutic target.

#### MiR‐34a

Expression of miR‐34a is decreased in many cancers, including prostate, non‐small‐cell lung, colorectal and pancreatic cancers 23. MicroRNA‐34a mediates p53 action on growth arrest, senescence, and apoptosis, as well as inhibition of epithelial‐mesenchymal transition.

MicroRNA‐34a was found to function as a novel node in the squamous cell differentiation network. The expression of miR‐34a increased with keratinocyte differentiation and decreased in skin and oral SCCs, SCC cell lines, and aberrantly differentiating primary human keratinocytes 24. In addition, SIRT6 (sirtuin‐6) was proved to be the direct target and determinant of its impact on differentiation. SIRT6 expression level is inversely correlated with miR‐34a in normal keratinocytes and keratinocyte‐derived tumours. SIRT6 plays a significant role in DNA repair and genomic stability and has been demonstrated to act as a tumour suppressor in liver and colorectal cancers. Since SIRT6 was up‐regulated in cSCC tissues and cell lines, it might play an opposite and/or more complex function in keratinocytes differentiation. Furthermore, miR‐34a could induce differentiation of human keratinocytes both *in vitro* and *in vivo*.

Taken together, the role of miR‐34a‐SIRT6 axis might help to understand the pathogenesis of cSCC, which is characterized by abnormal differentiation. In addition, it may also contribute to developing useful biomarkers for treatment of premalignant and malignant SCCs and beyond.

#### MiR‐124/214

MicroRNA‐124 (also called miR‐124a) plays a significant role in gastrulation and neural development 25. Deregulated miR‐124 is described to play important roles in different types of cancer. Through suppressing various genes, miR‐124 can suppress cancer growth and metastasis in many cancers, including colorectal cancer, breast cancer, hepatocellular carcinoma, gastric cancer and prostate cancer 26, 27, 28, 29, 30. MicroRNA‐214 can operate in opposite manners in different types of tumours. In some cancers, miR‐214 plays oncogenic role, while in others cancers suppressive role. High miR‐214 expression is observed in many cancers including pancreatic, prostate, stomach, nasopharyngeal, and lung tumours, as well as in some kinds of cutaneous T‐cell lymphomas. Conversely, miR‐214 downregulation occurs in hepatocellular, bladder, cervical, and colorectal carcinomas 31. The complexity of miR‐214 function depends on different targets and signalling, which granted the future investigation in different context.

Yamane *et al*. showed that miR‐124 and miR‐214 was decreased in cSCC both *in vitro* and *in vivo* 32. Supplement expression of miR‐124 and miR‐21 inhibited the cell proliferation *via* normalizing ERK1/2 (extracellular regulated protein kinases) levels in the cSCC cell *in vitro*. Additionally, serum concentration of miR‐124 was inversely correlated with cSCC progression. Overexpression of miR‐124 decreased expression of ERK2 protein, indicating that ERK2 was the target of miR‐124. However, transfection of miR‐214 mimic reduced the expression of both ERK1 and ERK2, suggesting that ERK1 and ERK2 was the target of miR‐214.

In conclusion, the down‐regulation of miR‐124/‐214 increased ERK1/2 expression, which subsequently mediates the hyperproliferation of cSCC tumour cells. The role of miR124/214 in the abnormal keratinocyte proliferation may lead to the development of useful targets for early diagnosis and novel treatment.

#### MiR‐125b

MicroRNA‐125b is implicated in multiple human cancers; however, the role of miR‐125b in cancer remains controversial. In some cancers, it is up‐regulated while in others it is downregulated. For example, the increased expression of miR‐125b was observed in breast cancer, oral SCC, and bladder cancer, in which miR‐125b inhibits tumour growth *in vitro* and *in vivo* 33, 34, 35, 36, 37. Meanwhile, decreased expression of miR‐125b was observed in prostate cancer, in which it can promote growth of prostate cells 38.

Comparing with healthy skin, miR‐125b was found to be down‐regulated in cSCC. Functional analysis revealed that miR‐125b suppressed proliferation, colony formation, migratory, and invasive capacity of cSCC cells *in vitro*. Furthermore, matrix metallopeptidase 13 (MMP13) was proved to be a direct target of miR‐125b since their expression levels were inversely related in cSCC 39.

To conclude, these findings indicate that miR‐125b plays a critically suppressive role in cSCC by targeting MMP13. Previously study has reported the therapeutic potential of MMP13, indicating that overexpression of miR‐125b in cSCC cells could also be a potential therapeutic candidate in cSCC.

#### MiR‐155

MicroRNA‐155 is a critical regulator in the immune system and immune function of miR‐155‐knock‐out mice is impaired 40, 41, 42, 43. In addition, miR‐155 plays an oncogenic role in various cancers and it is overexpressed in many cancers 44, 45, 46. However, recent data have revealed that miR‐155 may also exhibit anti‐oncogenic properties and exhibit immunological prevention against cancers 47, 48. Therefore, the role of miR‐155 in cancer remains unclear.

High‐risk human papillomavirus (HPV), such as HPV16 and HPV18, lead to many squamous cells carcinomas, including virtually all cases of cervical cancer and a significant proportion of other anogenital cancers, as well as some head and neck squamous cancers 49. The K14‐HPV16 transgenic mouse model is often used to study the pathogenesis of HPV‐associated squamous cancers. Using this model, Paiva *et al*. showed that all transgenic mice with demonstrated persistent epidermal squamous hyperplasia or *in situ* carcinoma and hyperkeratosis while the wild‐type mice did not develop any skin lesion. In hyperplastic skin samples, miR‐155 expression was lower than in normal chest skin.

In summary, miR‐155 expression was lower in HPV‐induced hyperplastic skin, indicating that miR155 levels may exhibit immunological prevention against carcinogenesis induced by HPV16.

#### MiR‐193b/365a

Previous reports showed that miR‐193b and miR‐365 were decreased in melanoma and liver, lung and colon cancers 50, 51, 52, 53. Gastaldi *et al*. found that miR‐193b‐3p and miR‐365‐3p are highly expressed in epidermis. However, there is a parallel decrease of their expression during cSCC progression. Overexpression of these miRs reduced tumour cell proliferation, clonogenic potential and migration. Furthermore, KRAS (kirsten rat sarcoma viral oncogene) and MAX were identified as direct targets of miR‐193b and miR‐365a. The expression levels of KRAS and MAX were significantly decreased by miR‐193b‐3p and miR‐365‐3p 54.

MicroRNA‐193b and miR‐365 act tumour suppressor functions in cSCC, inhibiting skin carcinogenesis through repression of KRAS and MAX. Restoration of miR‐193b‐3p or miR‐365‐3p by delivery of these miRs could provide promising future strategies for cancer treatment.

#### MiR‐199a

The expression of miR‐199a was altered in various cancers 56, 57. For example, the miR‐199a was up‐regulated in ovarian cancer, gastric cancer and cervical carcinomas 57, 58, 59 while it was downregulated in liver, breast and bladder cancer 54.

Wang *et al*. showed that expression of miR‐199a was reduced in human cSCC tissues. In addition, miR‐199a inhibited the proliferation and migration of cSCC cells *via* directly targeting CD 44. Moreover, miR‐199a played a role in the regulation of the interaction between CD44 and Ezrin 60.

Therefore, the tumour suppressive role of miR‐199a in cSCC by targeting CD44 might be an adjuvant strategy for the treatment of cutaneous SCC.

#### MiR‐361‐5p

VEGFA is a homo‐dimeric heparin‐binding glycoprotein, which associated with several types of skin cancer 61, 62, 63, 64. In particular, elevated levels of VEGFA were observed in cSCC 63. Kanitz *et al*. found that miR‐361‐5p suppressed the expression of VEGFA *in vitro* and were inversely correlated with VEGFA expression in cSCC and in healthy skin. In addition, miR‐361‐5p levels were decreased in cSCC compared to healthy skin, indicating that miR‐361‐5p might affect cSCC progression by regulating VEGFA in cSCC 55.

In conclusion, miR‐361‐5p might represent as a diagnostic and prognostic marker in cSCC. Further studies with larger sample numbers and follow‐up data are needed.

#### MiR‐483‐3p

The pathological implication of miR‐483‐3p in the development of tumours remains not controversial. Reduced levels of miR‐483‐3p are observed in some cancers, including gastric, pancreatic, and hepatocellular carcinomas and it could has anti‐tumour properties in those cancers 65. Meanwhile, miR‐483‐3p is up‐regulated and plays an oncogenic role in some tumours 66, 67.

Bertero *et al*. showed that miR‐483‐3p had pro‐apoptotic propertieson cSCC cells, through targeting various anti‐apoptotic genes, such as *API5, BIRC5* and *RAN*. Furthermore, an *in vivo* subcutaneous delivery of miR‐483‐3p significantly inhibited tumour growth SCC xenografts. MicroRNA‐483‐3p had a tumour suppressor function on cSCC by sensitizing tumour cells to apoptosis 68. The intra‐tumoural delivery of miR‐483‐3p provides an option for treatment of cSCC.

### MiRs up‐regulated in cSCC

#### MiR‐21

MicroRNA‐21 (miR/miR‐21), up‐regulated in various cancers, is a well‐established oncogenic miR 69, 70, 71, 72, 73, 74. MicroRNA‐21 expression was also increased in cSCC 39, 75. In addition, increased levels of miR‐21 are associated with reduced expression of tumour suppressors GRHL3 and PTEN 76. Mice subcutaneously injected with transformed keratinocytes lacking Grhl3 demonstrated increased tumorigenesis, suggesting that decreased Grhl3 expression contributes to tumour progression and up‐regulation of the oncomir miR‐21 in squamous cell carcinoma of the skin. Another study also showed that miR‐21 downregulated the expression of tumour suppressor genes, PDCD4 and PTEN 77. Downregulation of miR‐21 inhibited cell growth and invasion and induces apoptosis of cSCC cells. Therefore, ASO‐miR‐21 may have potential applications as a therapeutic target.

Understanding the role of miR‐21 and its target gene in cSCC may help to understand the pathogenesis of cSCC. Furthermore, gene therapy targeting miR‐21 should be further investigated to explore novel therapeutic candidate for cSCC.

#### MiR‐31

MicroRNA‐31 is frequently deregulated miRs in human cancers 78, 79, 80. However, its role in tumour development is remains not fully understood. MicroRNA‐31 can behave as either as a tumour suppressor or an oncogenic miR since it is up‐regulated in head and neck squamous cancer, liver cancer and colorectal cancer, but downregulated in gastric cancer breast cancer and prostate carcinoma 81. MicroRNA‐31 can regulate cell growth, migration, metastasis in cancers.

MicroRNA‐31 expression was higher in cSCC than healthy skin and precancerous skin lesions 39, 82. In addition, miR‐31 was specifically up‐regulated in cSCC cancer cells. Downregulation of endogenous miR‐31 suppressed migration, invasion and colony forming ability in human metastatic cSCC cells.

In conclusion, miR‐31 acts an oncogenic role in cSCC. Treatment targeting miR‐31 may provide a novel therapeutic method for cSCC.

#### MiR‐205

MicroRNA‐205 was overexpressed in head and neck SCC cell lines. In addition, SHIP2 (Src homology 2‐containing 5'‐inositol phosphatase 2) levels were inversely correlated with miR‐205 and suggest that high levels of miR‐205. Antagomir to miR‐205 increased apoptosis *via* restoring the expression of SHIP2 in SCCs 83. Therefore, antagomir to miR‐205 may provide a novel treatment of cSCC.

#### MiR‐365

Deregulation of miR‐365 is reported in many kinds of cancer. For example, miR‐365 is overexpressed in human breast cancer and it is also involved in the carcinogenesis of small cell lung cancer and colorectal cancer 52, 53.

MicroRNA‐365 was one of the highest expressed miRs induced by UVB treatment 84. In addition, Zhou *et al*. showed that miR‐365 was up‐regulated in both cells and clinical specimens of cSCC and it promoted the development of cSCCs through targeting nuclear factor I/B 85, 86. HaCaTpre‐miR‐365‐2 cell line, which overexpressed miR‐365, induced subcutaneous tumours while antagomir‐365, an anti‐miR‐365 oligonucleotide, inhibited cutaneous tumour formation *in vivo*. MicroRNA‐365 could also induce G1 phase arrest and apoptosis of cancer cells.

In summary, miR‐365 acts an oncogenic role in cutaneous SCC both *in vitro* and *in vivo*. The overexpression and roles of miR‐365 in cSCC indicate that it can be used as a potential indicator both in the clinical diagnosis and treatment.

## Conclusions

Cutaneous squamous cell carcinoma is one of the most common skin cancers with increasing number of new cases diagnosed each year. MicroRNA play a significant role in many physiological and pathological processes, especially in carcinogenesis. Increasing evidence has observed that deregulated miRs in cSCC, providing potential diagnostic and therapeutic targets. Anticancer cSCC treatment may be achieved by altering the deregulated level of miRs. Inhibitions of oncomiRs overexpressed in cSCC by administration of complementary nucleic acid sequences or by decreasing the actual expression level provides a potential therapeutic method. For example, inhibitory medicines against miR‐21s can be used in treatment of cSCC patients in future. However, further investigations are needed to confirm the therapeutic potential of miRs of cSCC clinically.

## Conflicts of interest

The authors have no conflicts of interest to disclose.
